# Selecting the embryo with the highest implantation potential using a data mining based prediction model

**DOI:** 10.1186/s12958-016-0145-1

**Published:** 2016-03-03

**Authors:** Fang Chen, Diane De Neubourg, Sophie Debrock, Karen Peeraer, Thomas D’Hooghe, Carl Spiessens

**Affiliations:** Leuven University Fertility Centre, UZ Leuven Campus Gasthuisberg, Herestraat 49, Leuven, Belgium; UZ Leuven Campus Gasthuisberg, Herestraat 49, Leuven, Belgium

**Keywords:** Prediction model, ART, Embryo selection, Implantation

## Abstract

**Background:**

Embryo selection has been based on developmental and morphological characteristics. However, the presence of an important intra-and inter-observer variability of standard scoring system (SSS) has been reported. A computer-assisted scoring system (CASS) has the potential to overcome most of these disadvantages associated with the SSS. The aims of this study were to construct a prediction model, with data mining approaches, and compare the predictive performance of models in SSS and CASS and to evaluate whether using the prediction model would impact the selection of the embryo for transfer.

**Methods:**

A total of 871 single transferred embryos between 2008 and 2013 were included and evaluated with two scoring systems: SSS and CASS. Prediction models were developed using multivariable logistic regression (LR) and multivariate adaptive regression splines (MARS). The prediction models were externally validated with a test set of 109 single transfers between January and June 2014. Area under the curve (AUC) in training data and validation data was compared to determine the utility of the models.

**Results:**

In SSS models, the AUC declined significantly from training data to validation data (*p* < 0.05). No significant difference was detected in CASS derived models. Two final prediction models derived from CASS were obtained using LR and MARS, which showed moderate discriminative capacity (c-statistic 0.64 and 0.69 respectively) on validation data.

**Conclusions:**

The study showed that the introduction of CASS improved the generalizability of the prediction models, and the combination of computer-assisted scoring system with data mining based predictive modeling is a promising approach to improve the selection of embryo with the highest implantation potential.

**Electronic supplementary material:**

The online version of this article (doi:10.1186/s12958-016-0145-1) contains supplementary material, which is available to authorized users.

## Background

There is a growing concern about the risks of multiple pregnancies. Worldwide, the incidence of multiple pregnancies has increased and the main reason for this increase is the use of assisted reproductive technologies (ART) [1]. These multiple pregnancies are leading to a higher incidence of pregnancy complications [2–6]. Single-embryo transfer (SET) after in vitro fertilization (IVF) is the only effective means to avoid multiple pregnancy in ART cycles. The success rate of SET is highly depending on the efficiency of the selection of the embryo with the highest implantation potential [7].

Ever since the introduction of ART, embryo selection has been based on the morphological characteristics of the cleavage stage embryo. At the early cleavage stage, the standard scoring system (SSS) based on the combination of several direct morphological parameters. Obviously an embryo has to reach a certain cell number at a given day, and can be further characterized by its morphological appearance based on the equal or unequal size of blastomeres and the degree of fragmentation. To date, cell number, proportion of fragmentation, symmetry of blastomere size, remains the main approach of embryo selection [8–12]. Several studies focused on this topic. Holte [10] showed that blastomere number, the degree of fragmentation, the size variation of blastomeres and the symmetry of embryos were independent predictors of implantation potential of the embryo. Cleavage rate was reported to clearly provide powerful prognostic information for implantation [8, 13]. Severe fragmentation of the embryo and uniformity in blastomere size were associated with poor prognosis [8, 13]. However, the presence of an important intra-and inter-observer variability in the microscopic evaluation [14, 15], and the absence of a clearly defined standard method, make it essential to further improve the selection techniques.

New imaging technology using multilevel images combined with a computer-assisted scoring system (CASS) has the potential to overcome most of the disadvantages associated with the standard scoring system (SSS). This computer-assisted scoring system allows switching focuses every 5 μm and stores multilevel images of embryos under inverted microscope, which allows further evaluation and measurement of the morphologic and morphometric characteristics of early cleaving embryos in a semi- automatic and more precise manner. Based on an objective measurement method, CASS overcomes the subjectivity of SSS, reporting less intra-and inter-observer variability [15]. Furthermore a significant association between total embryo volume on Day 2 and 3 and pregnancy rate was reported by Paternot [16]. Both lower and higher volumes were associated with a lower probability of successful pregnancy [16]. In addition, it has reported that CASS gave a better prediction of the implantation potential and live birth rate than SSS with multiple logistic regression based on morphologic characters on Day 3 [16]. Although this prediction model was not validated using an external validation data set.

Apart from morphologic embryo characteristics, several studies investigated the impact of clinical and diagnostic characteristics of the patients on the outcome of an ART cycle. Female and male age were reported to be associated with ART outcome [17, 18]. The impact of patient characteristics on ART outcome has been investigated such as the impact of endometriosis. One study concluded that ART yielded similar pregnancy outcome in patients with different stages of endometriosis when compared to patients with tubal infertility [19], while Azem et al. [20] reported that patient with stages III and IV endometriosis have a poorer outcome of ART than patients with tubal infertility. Nevertheless, there is still a lack of solid data on how to rank and select embryos based [21] on embryological data in combination with clinical, patient-related data.

To date, over 21 prediction models integrating multiple characteristics have been reported [21]. However, only 1/3 of the studied included embryo characteristic. And most of the models haven’t gone through an internal or external validation, which are essential to confirm the reproducibility and generalizability of a model process [21]. Only three models had a good performance after external validation [22–24]. Logistic regression is a generalized linear regression which has been the usual choice of method in binary medical events, such as mortality, pregnancy. Similar to linear regression, logistic regression may include only one or multiple independent variables the estimated probability of being in one binary outcome category versus the other, rather than representing an estimated continuous outcome [25]. To develop models with better discriminative capacity, a more advanced datamining method besides common used logistic regression method were implemented. Being different from logistic regression, which predefines a model, data mining methods learn a decision function by detecting underlying patterns without a predefined model. Next, the function is transformed into an objective function and then optimization methods are used to find the values of decision variables to reach the desired outcome with the most confidence. Data mining based clinical decision support systems are efficient tools that provide recommendations in medical diagnosis and treatment [26].

The first aim of this study was to construct a prediction model, using as well embryological data as clinical patient characteristics, for clinical pregnancy, with data mining approaches, and compare the predictive performance of models in SSS and CASS. We present a novel, intelligent, decision support system for IVF treatment, in view of the developmental and morphometric characteristics determined by a computer scoring system. This system is based on a multivariate adaptive regression splines (MARS) supervised embryo classification/ranking system that can assist on the selection of the most promising embryos for implantation in ART treatment. The second aim of the study was to compare selection made by SSS vs CASS.

## Methods

### Patients

Between January 2008 and December 2013, overall 1899 first ART cycles were performed with SET in women <36 years of age at the Leuven University Fertility Center resulting in 595 clinical pregnancies (31.3 %). Images were available of 871 transferred embryos, with 288 embryos (33.1 %) resulting in a clinical pregnancy. These embryos were evaluated by CASS, and further used for model construction.

Between January 2014 and June 2014, 192 first cycles with SET in women <36 resulted in 75 (39.1 %) clinical pregnancies. Of the 192 transferred embryos, 109 were recorded and evaluated by both SSS and CASS, consequently consisting the external validation set. Of the 109 transferred embryos, 42 embryos implanted resulting in a clinical pregnancy rate of 38.5 %.

Between January 2008 and June 2014, a number of 104 patients with at least two top quality embryos (eight cells on day 3, equally sized blastomeres, and ≤ fragments) were randomly recruited to compare the decision-makings of the current SSS based method and a prediction model assisted CASS method.

All procedures were in accordance with the Helsinki Declaration on Human Experimentation. The study was approved by the Commission for Medical Ethics of the University Hospital Leuven (approval reference number: ML4564).

### Ovarian stimulation and oocyte retrieval

The stimulation protocol used in this study has been published (Debrock 2010) [27]. Briefly, ovarian stimulation was carried out with gonadotropins (Menopur, Ferring, Copenhagen, Denmark; Gonal-F or Metrodin HP, Merck-Serono, Geneva, Switzerland; Puregon, Organon, Oss, The Netherlands) and GnRH agonists (Buserlin acetate, Suprefact; Hoechst, Frankfurt, Germany) during a long or short protocol. The follicular response was monitored by serum estradiol levels and transvaginal ultrasound measurements. The hCG, 10,000 IU, was administered when at least three follicles reached a diameter of 17 mm. Oocyte retrieval was performed 35 h after hCG injection by ultrasound guided transvaginal aspiration. The luteal phase was supported with intravaginal application of P (600 mg/day, Utrogestan; Besins, Drogenbos, Belgium) started at the evening of the hCG injection.

### IVF/ICSI procedures

After oocyte retrieval, oocytes were washed 4 times in order to minimize the amount of blood/follicular fluid and up to 5 oocytes were placed in a 4-well dish (Nunc, Thermo Fisher Scientific, Kamstrupvej, Denmark) containing GM501 Wash (Gynemed, Lensahn, Germany) under oil. Spermatozoa for the IVF/ICSI procedure were prepared using standard density gradient procedures (Isolate, Irvine Scientific, USA) or, in cases with very low sperm quality, diluted and centrifuged twice at 300 g for 10 min. For the IVF-procedure, the oocytes were transferred to a 4-well dish containing GM501 Culture (Gynemed, Lensahn, Germany) covered with mineral oil. Insemination was performed 2–6 h after oocyte retrieval. In the IVF procedure, oocytes were inseminated with 10,000 progressively motile spermatozoa per oocyte. In the ICSI procedure, the cumulus and corona cells were removed with hyaluronidase (conc.80 IU/m, Gynemed, Lensahn, Germany). The oocytes were injected with single sperm in a 20 μl droplet of medium. The injected oocytes were cultured individually in 20 μl culture medium (GM 501 Culture, Gynemed, Lensahn, Germany) droplets under oil. On Day 1 (16–20 h after insemination/injection) fertilization was evaluated. Only normally fertilized oocytes (two pronucleate, 2 PN) were cultured individually in a 20 μl droplet of culture medium (GM 501 Culture, Gynemed, Lensahn, Germany [16]) covered with mineral oil.

### Evaluation of fertilization, cleavage and embryo scoring

For both SSS and CASS, each embryo was checked, photographed and recorded within 1 min at the same time point. So there was no difference in the time of assessment between the two measurements. And in the lab, the time of assessment was fixed to a certain range, which minimized the impact of timing on embryos. In detail, multilevel images were recorded for each embryo on Day 1 (16–20 h after insemination/injection), Day 2 (41–44 h after insemination/injection) and Day 3 (66–71 h after insemination/injection, the day of transfer) using a computer system (CellCura Software Solutions, Copenhagen, Denmark). This system allowed switching focuses every 5 μm and presenting images of embryos in Z-stack.

The status of pronuclear of zygote on Day 1 was evaluated. The number of blastomeres, the size difference between blastomeres and the degree of fragmentation were evaluated on Day 2 and Day 3. With standard scoring, assessments were performed by the embryologist who visually evaluated the number and size variation of blastomeres and the degree of fragmentation on Day 2 and Day 3. The grading methods are further explained in variables section. With computer assisted scoring system, measurements were performed on the same series of images. The diameter of each blastomere was drawn manually, further morphometric characteristics: the total cytoplasmic volume (TCV), size difference and fragmentation were calculated precisely.

### Variables

To predict implantation potential, embryo characteristics and clinical factors of the couple were initially regarded as potential predictors. Class label was assigned as 1 for clinical pregnancy and 0 for no clinical pregnancy. A clinical pregnancy was defined as the presence of at least one intrauterine gestational sac with fetal heart beat at a gestational age of 12 weeks.

#### Clinical variables of the couple

The age of female and male, male pathology, female pathology (including ovulation disorders, the presence of endometriosis, transport problems, and implantation problems), type of infertility and duration of infertility were considered as clinical factors. Of these factors, age and duration of infertility were considered as numerical variables. Male and female pathology, ovulation disorders, transport problems, implantation problems, were considered as nominal variables, where 0 was used for absence and 1 was used for presence. The presence of endometriosis was considered as ordinal variables, where 5 levels were established: 0 = absence; 1 = stage I; 2 = stage II; 3 = stage III; 4 = stage IV.

#### Morphological embryo variables

The following morphological embryo variables were scored and recorded: number of blastomeres, size difference between blastomeres and degree of fragmentation. To be detailed, assessments were performed by the embryologist who visually evaluated the number and size variation of blastomeres and the degree of fragmentation on Day 2 and Day 3. The degree of fragmentation was divided into five categories: 0 = no fragmentation; 1 = 0–10 %; 2 = 10 %–20 %; 3 = 20 %–50 %; 4 = more than 50 % fragmentation. Symmetry score was graded into three levels: 0 = symmetrical blastomeres; 1 = slightly unequal blastomeres (25–50 % difference); 2 = uneven blastomeres (more than 50 % difference). Of these variables, size difference and the degree of fragmentation were considered as ordinal variables. Number of blastomeres was considered as numerical variable. In addition, a higher clinical pregnancy rate was observed to be associated with even numbers of blastomeres on Day 2. Thus, the presence of an even number of blastomeres on Day 2 was initially included as a nominal predictor.

#### Morphometric embryo variables

Morphometric embryo variables included the total cytoplasmic volume (TCV), the coefficient of diversity (COD) of size, and the percentage of fragmentation in CASS. In detail, based on the image sequences (Z-stack), diameters of the fertilized oocyte on Day 1 and individual blastomeres of embryos on Day 2 and Day 3 were drawn manually. The criteria for distinguishing between a blastomere and fragment was based on the findings by Hnida [28] and Johansson [29] who reported that the diameter of a blastomere should be ≥45 μm on Day 2 and ≥40 μm on Day 3. Based on the diameters, The coefficient of diversity (COD) defined as the size ratio of the largest/smallest blastomere, was calculated. The total cytoplasmic volume (TCV) of the fertilized oocyte on Day 1 and the total volume of the blastomeres of embryos on Day2 and Day 3 were calculated automatically. Based on the principle that the total volume does not change during the first days of development, the reduction of TCV on Day 2 and Day 3 from Day 1 was calculated and interpreted as fragmentation.

#### Comparison of both scoring systems

In addition to the evaluation of discriminative capaticty of both scoring systems, a retrospetive comparison was made between the decisions made by SSS and CASS. A dataset comprised patients underwent SET who had more than one top quality embryos (8 cells on Day 3, equal or slightly unequal sized blastomeres, less than 10 % fragmentation) sored by SSS was initially generated. Then 104 patients were randomely selected as a validation cohort. The control group consisted of the transferred embryos in lab which were selected based on SSS. In the study group, for each patient, the top quality embryos were reevaluated by CASS and then went through with the prediction model, obtaining the embryo which should be transferred according to the CASS based model. The decisions of SSS and CASS were compared.

### Statistical analysis

We calculated intra-class correlation coefficient (ICC) and Cohen’s Kappa coefficient to measure intra-observer agreement in continuous and categorical variables respectively. It was interpreted as an indicator of either excellent (≥0.8), good (0.60–0.79), moderate (0.40–0.59), poor (0.20–0.39) and very poor (<0.20) intra-agreement.

Among the clinical variables, maternal age was considered as potential confounding variables. The maternal age between the two groups were compared. We carried out a Kolmogorov–Smirnov test, which was sensitive to both location and shape, to assess the significance of difference in distributions.

Since collinearity between independent variables complicate or prevent the identification of an optimal set of explanatory variables for a statistical model, we perform a variance inflation factors (VIF) analysis to identify the collinearity among explanatory variables. We used function “collidiag”, which is available and explained in detail on the internet: https://github.com/brian-lau/colldiag. Briefly, the input argument is a given matrix, and the function retains the variance inflation factors. Given a design matrix, the condition indices (ratio of largest singular value to each singular value), variance decomposition proportions, and variance inflation factors are returned. A univariate logistic regression was performed for each clinical variable in variable selection (Table 1). Although hypothesis test is usually performed with a significance level of 0.05, a different significance level for variable selection in model building is commonly used, as the incorrect exclusion of a factor would be more deleterious than the inappropriate inclusion a factor [30]. In this study, a less stringent p-value (*p* < 0.3) was used to ensure evaluation of a wide range of potential predictors. To ensure the accuracy of the final model, “transport problem” was excluded because of a high number of missing values (e.g. this aspect was quite often not investigated in case of male factor problems).

**Table 1:** Univariate logistic regression analysis of clinical characteristics

Features	β	OR	95 % CI	*p*- value
Female pathology	−0.08	0.92	0.68–1.27	0.62
Ovulation	0.08	1.08	0.79–1.49	0.63
Endometriosis	−0.04	0.96	0.83–1.12	0.98
Transport	−0.42	0.66	0.38–1.16	0.12
Implantation	−0.04	0.96	0.57–1.64	0.88
Male pathology	0.04	1.04	0.76–1.42	0.93
Age of male	−0.03	0.97	0.95–1.00	0.05
Age of female	−0.03	0.97	0.93–1.01	0.11
Type of infertility	−0.14	0.87	0.61–1.23	0.43
Duration of infertility	0.00	1.00	0.99–1.01	0.51

*OR* odds ratio

*CI* confidence interval

*β* beta coefficient

Analysis with spline functions indicated a nonlinear association between number of blastomeres on Day 3 and implantation rate, hence a univariate quadratic regression model was used prior to multivariate regression. According to the quadratic regression equation, the theoretic best number of blastomeres on Day 3 should be 8.4. (Additional file 1: Figure S1) To fit the model better, number of blastomeres on Day 3 was transformed by taking the absolute value of the deviation from 8.4, e.g. an embryo with 8 blastomeres on Day 3 was recoded as 0.4 (8 minus 8.4).

Logistic regression (LR): Logistic regression has been the most commonly used method for predicting binary outcomes in medical research. In this study, the LR models were constructed using backward variable elimination. A model consisted of all the features, allowing two-way interaction between variables was primarily obtained. All of the variables may not have a significant prognostic effect on implantation outcome. Then, the final multivariate model was obtained by a stepwise procedure, where variables were excluded at each step if their removal didn’t reduce the predictive outcome of the model significantly (*p* < 0.1). The analysis was carried out using functions in toolbox of GeneralizedLinearModel implemented in MATLAB (2014a) http://nl.mathworks.com/help/stats/generalized-linear-regression-1.html.

Multivariate adaptive regression splines (MARS): MARS is a flexible, nonparametric regression modeling technique that requires no assumptions about the relationships among the variables. It constructs a relation between dependent and independent variables from a set of coefficient and basic functions, which are evaluated at different knot values. MARS generates a model in a two-stage process: a forward pass and a backward pass. The forward pass creates a model which is usually overfitting, the backward pass prunes the model by removing less effective basic functions based on generalized cross validation (GCV) criterion. Our MARS model was built through 10-fold cross validation, which separated the dataset into 10 equal sized parts, fit a model with 9 parts and calculated the prediction error on the 10th part. This procedure was performed 10 times and calculated the average of prediction errors. The toolbox to develop a MARS model implemented in MATLAB was ARESLab toolbox (Jekabsons G., ARESLab: Adaptive Regression Splines toolbox for Matlab/octave, 2011, available at http://www.cs.rtu.lv/jekabsons/).

To evaluate the performance of the proposed model, receiver operating characteristic (ROC) analysis was employed. This statistical analysis was carried out using function of “perfcuv” in MATLAB. The ROC curve plots the sensitivity versus 1-specificity by adjusting the decision threshold of classification. Area under the curve (AUC) was calculated. An area of 1 represents a perfect system; an area of 0.5 represents a worthless system (the same result can be obtained by randomly selecting the output class). A rough guide for evaluating the accuracy of a system is the traditional academic point system: 0.9–1 = excellent; 0.8–0.9 = good; 0.7–0.8 = fair; 0.6–0.7 = poor; 0.5–0.6 = fail. Finally, the AUC were compared to present the difference of accuracy of each model using pROC R-package in R environment [31, 32]. It retained the *p*-value as the difference between groups. A true difference in AUC is equal to 0.

## Results

Among the clinical variables, maternal age was considered as potential confounding variables. The returned value of h = 0 indicated that ks-test did not reject the null hypothesis that the studied factors were from the same continuous distribution. In univariate analysis of clinical variables, age of female and male were found to be associated with implantation (Table 1).

Prior to development of the predictive model, the intra-observer reliability of computer-assisted scoring system was evaluated. An excellent intra-observer agreement for the CASS was found for the evaluation of features on Day 1 and Day 2, and number of blastomeres on Day 3. The intra-observer agreement was good for the evaluation for the other characteristics on Day 3 (Table 2).

**Table 2:** Intra-observer agreement in embryo evaluation performed on CASS

Period	Characteristics	Correlation coefficient
Day1		
	Total Cytoplasmic Volume	0.974*
Day2		
	Total Cytoplasmic Volume	0.960*
	Number of Blastomeres	0.986*
	Percentage of Fragmentation	0.956*
	Size difference between Blastomeres	0.967*
Day3		
	Total Cytoplasmic Volume	0.782*
	Number of Blastomeres	0.986*
	Percentage of Fragmentation	0.789*
	Size difference between Blastomeres	0.747*

*p < 0.05

Descriptive analyses were performed on morphometric parameters of embryos from different categories. Firstly, the COD of embryos with different number of blastomeres were compared both on Day 2 and Day 3 (Table 3). On Day 2, the COD among embryos in different cell stage showed a significant difference. On Day 3, only 8 cell stage embryos showed significant (*p* < 0.05) difference when compared with other cell stages. Secondly, the COD of embryos that, implanted or did not implant were compared on day 3. A significant difference (*p* < 0.05) was only found in 8 cell stage embryos on Day 3 (Table 4), where implanted embryos were observed to have equally sized blastomeres. Thirdly, the correlation between TCV and degree of fragmentation was also investigated. As indicated in Fig. 3, total embryo volume was significantly negatively correlated to the degree of fragmentation.

**Table 3:** Coefficients of diversity (ratio of largest/smallest blastomere: mean ± SD) for human embryos

Day	Blastomeres (n)	Coefficient of diversity
Day 2	2	1.096 ± 0.015*
	3	1.310 ± 0.184*
	4	1.206 ± 0.122*
	5	1.385 ± 0.164*
	6	1.513 ± 0.175*
Day 3	6	1.363 ± 0.152
	7	1.352 ± 0.160
	8	1.260 ± 0.119*
	9	1.365 ± 0.136
	10	1.354 ± 0.106

*represented significant difference compared with all the other categories on the same age(*p* < 0.05)

**Table 4:** Coefficient of diversity (ration of largest/smallest blastomere: mean ± SD) for embryos on Day3

Blastomeres (*n*)	Number of observations	Non-implanted	Implanted	*p*-value
6	58	1.356 ± 0.158	1.389 ± 0.123	0.511
7	156	1.359 ± 0.157	1.324 ± 0.167	0.251
8	496	1.276 ± 0.131	1.244 ± 0.104	0.004
9	106	1.383 ± 0.150	1.334 ± 0.095	0.161
10	29	1.395 ± 0.044	1.354 ± 0.118	0.381

**Fig. 1:**
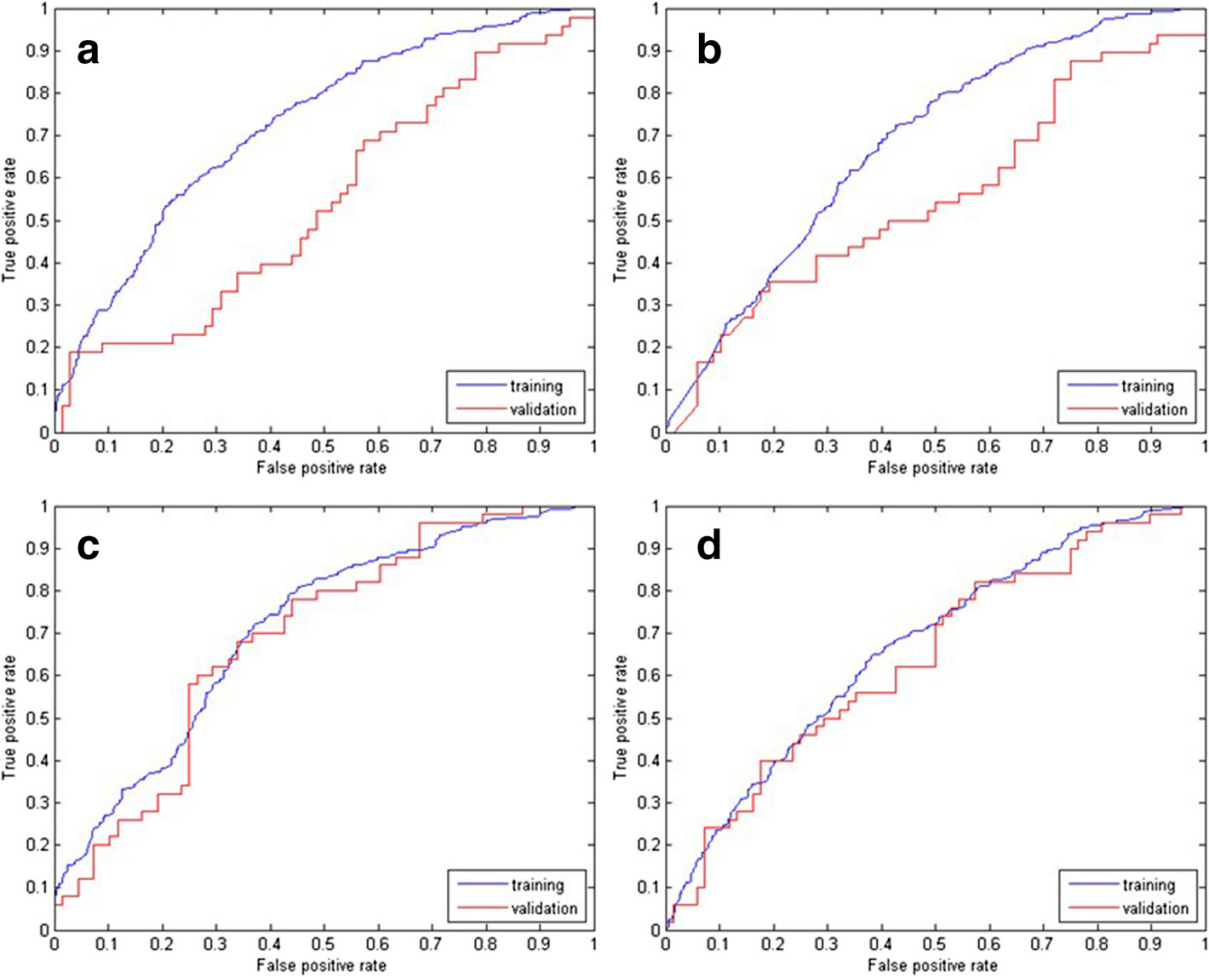
ROC curve for classification by MARS and LR with of scoring system. **a**: MARS model of SSS (*p* = 0.02); **b**: LR model of SSS (*p* = 0.03); **c**: MARS model of CASS (*p* = 0.71); **d**: LR model of CASS (*p* = 0.64)

The area under ROC curve (AUC) for each model in both training set and validation set is reported in Table 5 and Fig. 1. The area under the ROC curves were compared using “pROC” implemented in R. With CASS, the AUC for the LR model decreased from 0.67 in the training set to 0.64 (*p* = 0.64) in the validation samples. Similarly, the decline in ROC curve area between the training set and validation set negligible for the MARS model: from 0.71 to 0.69 (*p* = 0.71). For the models based on standard scoring system a greater decline was found. The ROC curve areas for the logistic regression models were significantly different, from 0.68 in training set to 0.55 in validation set (*p* = 0.03). For the MARS model, the AUC dropped from 0.73 to 0.54 significantly (*p* = 0.02). The decreases in AUC of on SSS datasets were indicative of a tendency of both models to be over-fitted on the training samples.

**Table 5:** The discrimination power of predictive models

Measure Method	Data Set	LR model	MARS model
CASS	Training	0.67	0.71
	Validation	0.64	0.69
SSS	Training	0.68	0.73
	Validation	0.55	0.54

Detailed information of models based on CASS is presented in Table 6. In view of both training data and external validation data, the MARS model resulted in a significant (*p* = 0.02) better discrimination accuracy in training data (0.71) compared with LR model (0.67). However, the difference in AUC (0.69 and 0.64, respectively) in validation data was not significant (*p* = 0.30). The LR model consisted of 8 predictors: number and parity of blastomeres on Day 2, morphometric parameters (TCV, fragmentation and COD) on Day 2, number of blastomeres and COD on Day 3, and paternal age. Total cytoplasmic volume on Day 1 and Day 3, age of female were not significant and subsequently excluded. And an equation predicting the implantation occurrence was obtained in Table 7. The MARS model consisted of 22 basic functions, including all of the 12 predictors. The parameters were trained by the function of “areasparams” in ARESLab. The maximum number of basic functions included in model in the forward building phase was calculated automatically using formula min(200, max(20, 2*d*)) + 1, where *d* is the number of input variables according to user’s manual. The GCV penalty per knot was 3, as commended by Friedman [33, 34]. The rest parameters, whether to use piecewise-cubic type modeling, the level of piecewise-cubic modeling, threshold, prune, were determined by default according to user’s manual.

**Table 6:** Prediction models on CASS evaluations

Characteristics	Logistic Regression	MARS
Number of Basis Functions	11	22
Number of Predictors	8	12
Predictors on Day 1		
TCV	-	+
Predictors on Day 2		
Blastomere number	+	+
Status of parity	+	+
TCV	+	+
Fragmentation	+	+
Blastomere size difference	+	+
Predictors on Day 3		
Blastomere number	+	+
TCV	-	+
Fragmentation	-	+
Blastomere size difference	+	+
Age_of male	+	+
Age_of female	-	+

+ represented included predictors; - represented excluded predictors

**Table 7:** Logistic regression model of implantation potential

Predicter	Estimate	SE	t-Statistic	*p*Value
(Intercept)	12.070	4.446	2.715	0.007
Number_Day2	0.817	0.232	3.522	0.000
TCV_Day2	−1.572	0.547	−2.875	0.004
Fragmentation_Day2	−12.250	6.392	−1.917	0.055
Age_male	−0.311	0.134	−2.328	0.020
Number_Day3	−2.697	0.853	−3.164	0.002
Number_Day2:COD_Day3	−0.412	0.161	−2.549	0.011
TCV_Day2*Age_male	0.045	0.016	2.806	0.005
COD_Day2:Age_male	−0.065	0.026	−2.457	0.014
Fragmentation_Day2*Age_male	0.327	0.190	1.722	0.085
COD_Day2:Number_Day3	1.434	0.637	2.249	0.024
Parity_Day2:Number_Day3	0.730	0.225	3.246	0.001

Log(potential) = 1+ Number_Day2+ Number_Day3+ Number_Day2:COD_Day3 + TCV_Day2*Age_male + COD_Day2:Age_male + Fragmentation_Day2*Age_male + COD_Day2:Number_Day3+ Parity_Day2:Number_Day3

*Estimate* the estimated value for each coefficient

*SE* standard error for the coefficient estimate

To compare the decision-makings of current embryo selection criteria and the model assisted method, 104 patient were randomly recruited. Thirty-six (34.6 %) of 104 cases retained to a same decision, while in 68 (65.4 %) cases the transferred embryos of current criteria were not the best option according to our prediction model.

## Discussion

There is an increasing interest in using statistical methods to predict ART outcome in clinical research. In the current study, we have developed an approach to rank embryos according to their implantation potential taking into account both embryological and clinical features. The proposed model is expected to assist embryologists to determine which embryo to transfer. The model established a moderate discriminative performance, both in the training set and in the separate validation set. Our data also suggested that prediction models based on computer assisted scoring system using morphometric data, rather than standard scoring system, had significantly more stability and reliability of predictive capability. The TCV from Day 1 to Day 3, number of blastomeres, COD and fragmentation on Day 2 and Day 3 are considered as important embryo features to evaluate the implantation potential.

**Fig. 2:**
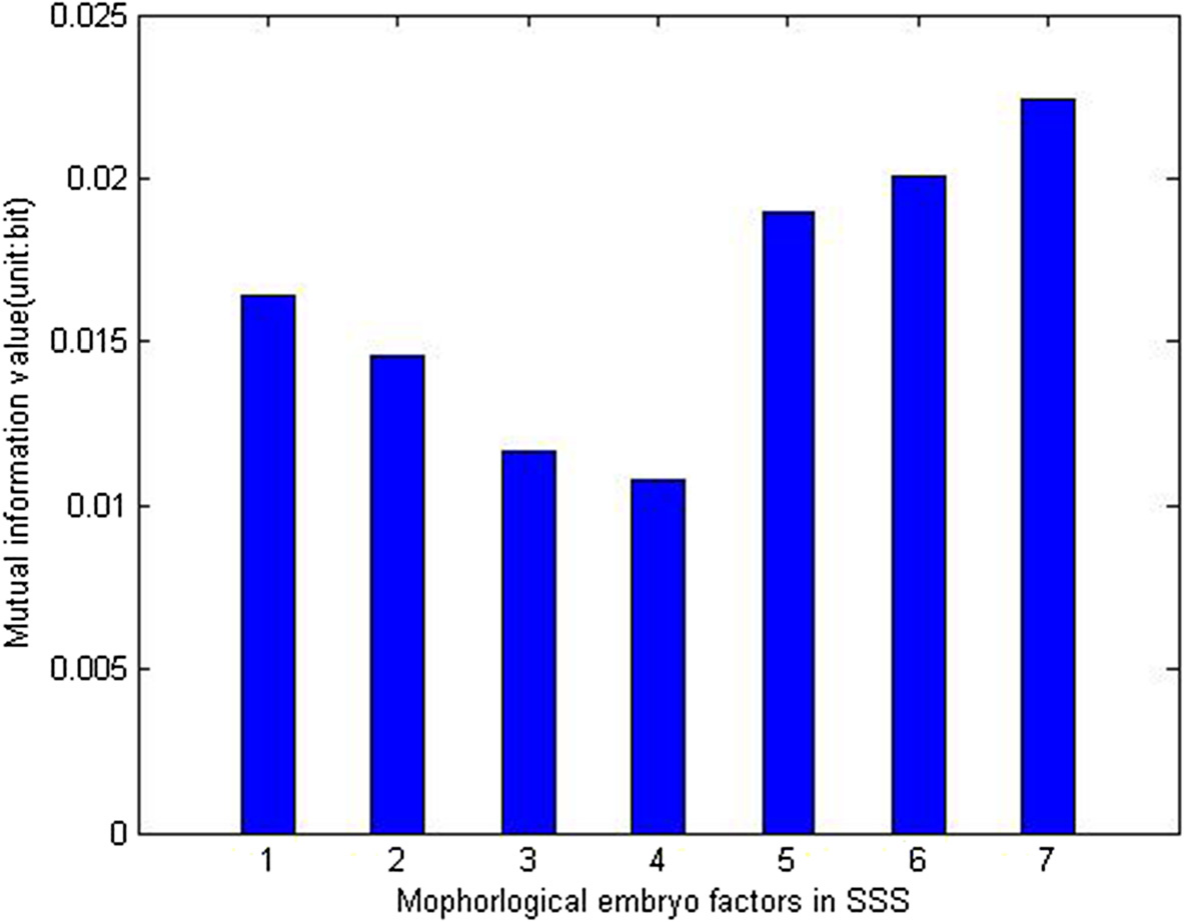
Mutual information analysis of correlation between characteristics and outcome. Each bar represents the correlation power of corresponding characteristic. 1: number of blastomeres on Day 2; 2: fragmentation on Day 2; 3: size difference between blastomeres on Day 2; 4: even or uneven blastomere number on Day 2; 5 number of blastomeres on Day 3; 6: fragmentation on Day 3; 7: size difference between blastomeres on Day 3. Embryo characteristics on Day 3 were more important to predict the implantation outcome compared with characteristics on Day 2

It was indicated that a CASS may be superior to a SSS in the prediction of implantation and live birth [16]. To further refine and validate, using an external validation dataset, the model proposed by Paternot et al. [35], we decided to use logistic regression and multivariate adaptive regression splines in both SSS data and CASS data, to develop prediction models. For each of the four models retained, the AUC of training data and validation data were compared [31, 32] to evaluate the generalizability. In the two models derived from SSS data, we found a significant decline in prediction accuracy in the external validation data set, suggesting that both LR model and MARS model based on SSS had poor predictive capability and considerable variation. Models using CASS data showed a more stable discriminative capability. According to mutual information analysis (Fig. 2) which reflect the correlation between variables, morphological characteristics on Day 3 are supposed to be the most powerful embryo features to determine treatment outcome. Consequently, low correlation coefficients of evaluations on Day 3 [15] may result in the poor performance of the external validation data set. In the CASS dataset, the MARS model presented a better predictive power compared with LR model. Results of univariate analysis have showed that not all of the characteristics were linearly correlated to clinical pregnancy, such as number of blastomeres and COD on Day 3. In such cases, MARS model, regardless of the certain assumption about the underlying functional relationship between the dependent and independent variables, constructed the relation from a set of coefficients and basic functions that are entirely “driven” from the data itself and consequently resulted in better AUC [36].

Number of blastomeres on Day 2 and Day 3 were found to be a powerful predictor clinical pregnancy. On Day 2, the presence of 4 blastomeres was considered the ideal cleavage rate [10, 37]. The study by Van Loendersloot et al. [24] showed that faster cleaving embryos had a lower chance of implantation. However, in our dataset, the presence of 6 blastomeres on Day 2 resulted in a higher implantation rate (42.1 %) than 4 blastomeres (39.7 %) but these results have to be further confirmed in future studies because only 19 embryos at the 6-cell stage on Day 2 were transferred, with 8 embryos implanted. The ideal number of blastomeres on Day 3 was 8. A marked reduction in implantation rate was found to be associated with slow cleavage rate and a slight reduction was correlated with rapid cleavage rate. This finding corroborates other studies where numerous authors have reported that too slow or too fast embryo cleavage has a negative impact on implantation rate [8, 13, 38]. This non-linear relationship between number of blastomeres and IVF/ICSI outcome makes the transformation (abs(number-8.4)) essential model fitting.

The study showed that the difference in blastomere size had strong predictive power for implantation. This is in line with other predictive models [39]. We compared coefficient of diversity (COD) of synchronously divided embryos (2-, 4- or 8-cell stage embryos) to embryos in intermediate steps (3-, 5-, 6-, 7-, 9- or 10-cell embryos). Significant lower COD was observed to be associated with synchrony. This finding confirmed a previous study [35], where the lowest coefficient of diversity was found for 8-cell stage embryos on Day 3. The variation in blastomere size has been reported to be negatively correlated with implantation [38]. In our study, implanted embryos tended to have more uniform blastomere size than non-implanted embryos except for 6-cell stage embryos, although significant difference was only found in 8-cell embryos on Day 3. It also confirmed the results described by [40] and the division model published by Roux [41] that theoretically, 6-cell stage embryos should have unequally sized blastomeres and higher COD.

Fragmentation on Day 2 and Day 3 were indicated as independent significant predictors of implantation in the multivariate models by Van Loendersloot [24] and Holte [37], respectively, and this was confirmed in both the LR and MARS model.

**Fig. 3:**
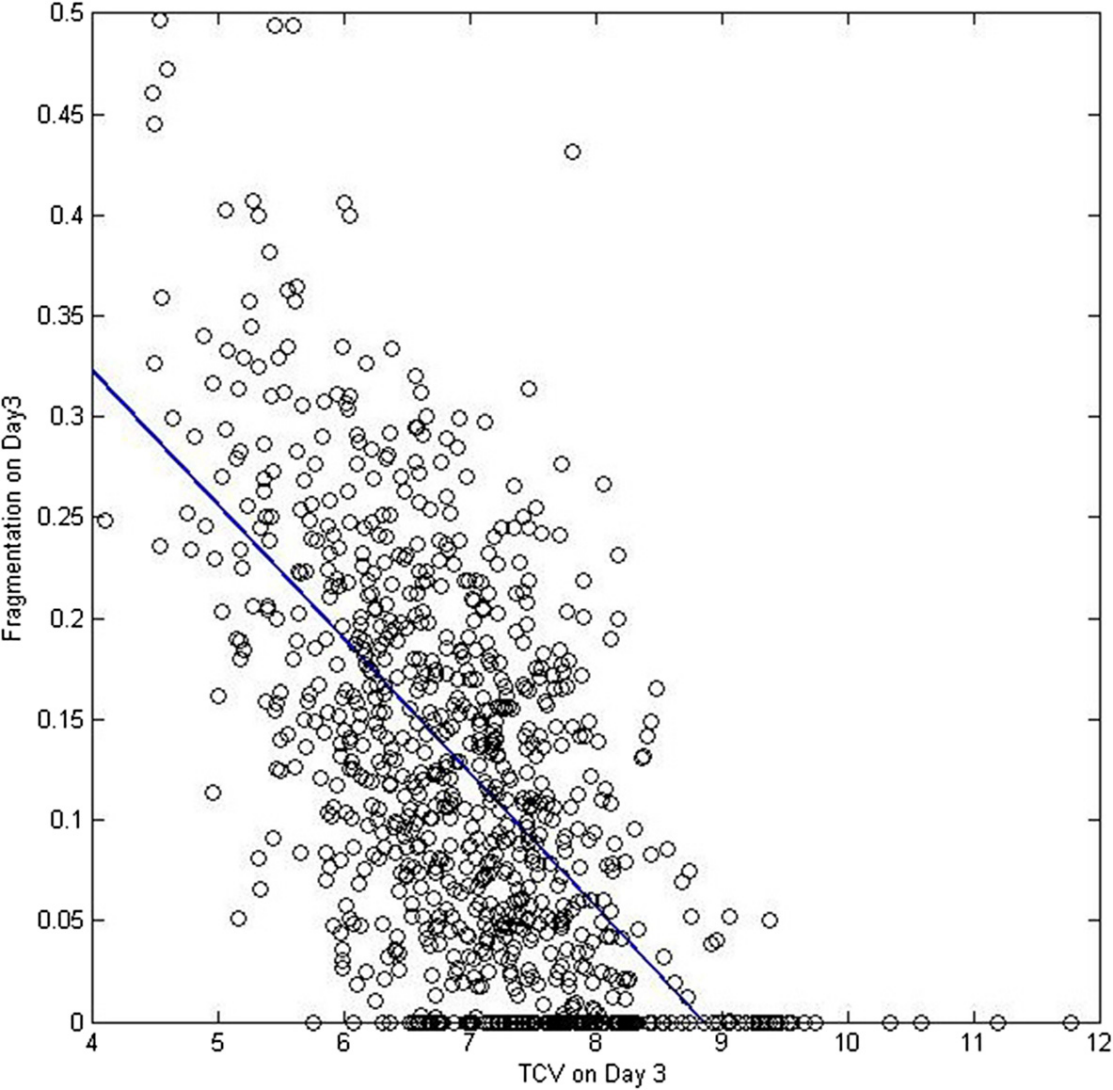
The correlation of fragmentation and total cytoplasmic volume. Total embryo volume was significantly negatively correlated to the degree of fragmentation

Total cytoplasmic volume on Day1, Day2 and Day 3 were all included in the final MARS model while only TCV on Day 2 was included in the LR model. An earlier study [42] identified a significant association between TCV and implantation potential on both Day 2 and Day 3. This could be explained by the fact that volume regulation is an essential process in the embryo development, as a failure in volume regulation can result in blocked embryos [43]. Hnida [44, 45] reported a significant decrease in the mean blastomere volume with an increasing degree of fragmentation for all analyzed embryo stages. Our current data analysis shows a significant (*p* < 0.01) negative correlation between the TCV and the degree of fragmentation (Fig. 3), confirming the conclusion published by Hnida [44, 45]. In addition, in the VIF analysis, a retained valued of 92 indicated a moderate to strong (30 ~ 100) collinearity between TCV and fragmentation on Day 3. The collinearity did not affect the final LR model since both predictors were excluded by stepwise logit regression. Nevertheless, the final MARS model, including both TCV and fragmentation fitted the data significantly better than the model including only one of the two parameters. Several other embryo evaluation models have been published in the past several years. However, some studies suffered from inadequate data sets and concluded their input information was not sufficient to classify the outcome [8, 10, 46]. Some other studies managed to achieve a model with high accuracy, without validation in an external data set, which is an essential process for a prediction model [10, 47]. The model by Van Loendersloot et al. [24] was based on the investigation of a large number of patients and cycles, and acquired confirmation on external validation data. However, when integrating number of blastomeres, degree of fragmentation, and size difference between blastomeres, the scoring method introduced in this study has limited prediction value for embryos with the same score but different fragmentation and COD status (e.g., an embryo with no fragmentation but unevenly sized blastomeres and an embryo with fragmentation but evenly sized blastomeres).

Several other morphometric characteristics have been considered as predictor for developmental competence or implantation, such as zygote size, nuclear size, embryo area and perimeter, equivalent circle radius of the embryo, embryonic roundness, and zona pellucida thickness [48–50]. Morphometric measurements may minimize the variability among different embryologists and clinics. Although very little relevant literature published on the predictive ability of morphometric parameters, further studies are worth to go. It may improve the understanding of basic biology controlling early embryonic development and how this is affected by clinical parameters.

According to our data, the type of female pathology and male pathology have limited influence on the success of IFV/ICSI treatment within the first cycle in univariate analysis. Notably, regardless of no significant difference on clinical pregnancy rate with the presence of endometriosis success rate in patient with severe endometriosis decreased remarkably in our research cohort. However, with the small population (49 in 871 cases), this factor did not contribute to the final model. Ages of male and female were interesting prognostic factors for implantation according to our models. Increasing age of both women and men has been reported to be associated with declining possibility of successful pregnancy chance [51–54]. As one of the most important factors for success with IVF, age of female is commonly included in previous prediction models. Male age, for the first time, is recruited in the prediction model in our study. Univariate logistic regression analysis revealed no significant influence of type of infertility and duration of infertility on clinical pregnancy, which is in line with Bancsi’ s [55] study.

The best predictive capacity of our cohort was 0.71 on training set and 0.69 on validation set. Compared with the previous prediction models, most of which could hardly return an accuracy rate of 0.67, this model presented a more confident prediction. Data trained for the model was collected during a period of 6 years, while the validation was performed on a separate and more recent data set. Regardless of different period, this model showed a similar discrimination efficiency on validation set. It should be noteworthy that all the data used in this study was collected from a single source, it is important to evaluate the generalizability of the model to other clinics. The method needs to be confirmed by replicating experiments on different IVF data sets.

It also should be noted that only single embryo transfers were taken into account when we developing the model. This approach has the advantage that the embryo data can be directly linked to the outcome. The major weakness of the study is the fact that the transferred embryos were selected using the SSS and the CASS analysis was performed retrospectively. In clinical single embryo transfer practice, embryologist are often confronted with situations that more than one embryo are available. In these cases, the CASS based scoring method may provide better understanding of all the characteristics of the embryo and assist the embryologist to select the embryo with highest implantation potential. The comparison research carried out on the 104 patients illustrated that in the majority of cases, this model assisted scoring method retained to a different decision, indicating that an evaluation of the this prediction model on future clinical randomized trial would be necessary. However, in the absence of a clinical randomized trial, this prediction model can be used to counsel couples undergoing ART treatment on the chance of a pregnancy, as predictions made by clinicians on the basis of clinical experience have only slight to fair reproducibility and in consequence a poor predictive accuracy [56].

## Conclusions

The results presented indicate a considerable variation in prediction accuracy of models based on standard scoring system. The significant decline in validation data compared with training data reveals a limited utility of prediction models in SSS. In comparison, computer-assisted scoring system and the related models present the advantage of less subjectivity and more generalizability.

In addition, the findings of this study show that combination of computer-assisted scoring system and data mining based prediction method has a promising benefit in the selection of best embryo to transfer in IVF/ICSI treatment. The results need to be confirmed in a prospective trial using the CASS for the final selection of the embryo to be transferred.

## Additional file

Additional file 1: Figure S1. Quadratic regression of number of blastomeres on day 3. (JPG 17 kb)

## Abbreviations

ART: assisted reproductive technologies
CASS: computer assisted scoring system
COD: coefficient of diversity
LR: logistic regression
MARS: multivariate adaptive regression spline
SET: single-embryo transfer
SSS: standard scoring system
TCV: total cytoplasmic volume
