# Supplementary figures and images for: Selecting the embryo with the highest implantation potential using a data mining based prediction model
Source: Reprod Biol Endocrinol. 2016 Mar 3;14:10. doi: 10.1186/s12958-016-0145-1 (PMC4776393; doi:10.1186/s12958-016-0145-1)

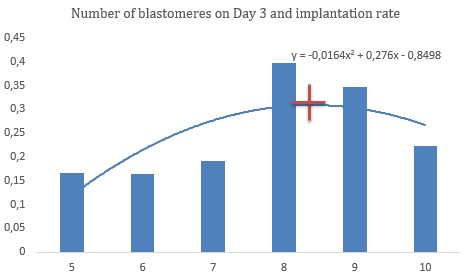

Supplement: Additional file 1: Figure S1. — Quadratic regression of number of blastomeres on day 3. (JPG 17 kb) [file 12958_2016_145_MOESM1_ESM.jpg]
